# Verification of automatic analysers Roller 20PN and iSED for measuring erythrocyte sedimentation rate

**DOI:** 10.11613/BM.2022.010708

**Published:** 2022-02-15

**Authors:** Helena Čičak, Pavica Šonjić, Ana-Maria Šimundić

**Affiliations:** 1 Department of Medical Laboratory Diagnostics, University Hospital “Sveti Duh”, Zagreb, Croatia; 2 Clinical Department for Laboratory Diagnostics, Rijeka Clinical Hospital Centre, Rijeka, Croatia; 3 Faculty of Pharmacy and Biochemistry, University of Zagreb, Zagreb, Croatia

**Keywords:** automated ESR, Westergren method, blood sedimentation rate, verification, haematology

## Abstract

**Introduction:**

Automated erythrocyte sedimentation rate (ESR) analysers are based on different methodology than Westergren method. It is questionable whether ESR values obtained from those analysers are comparable with determined values with Westergren method. The aim was verification of the precision, method comparison and accuracy of automated ESR analysers: Roller 20PN (Alifax S.p.A., Polverara, Italy) and iSED (Alcor Scientific, Smithfield, USA).

**Materials and methods:**

Blood samples (N = 752 for Roller 20PN and N = 213 for iSED) were sampled into K_2_EDTA (Kima, Italy) tubes for automated and 3.8% Na-citrate tubes (Kima, Italy) for Westergren method. The data was divided into three groups according to the ESR values obtained with the Westergren method: Group Low (L) (ESR ≤ 20 mm), Group Medium (M) (ESR 21-60 mm), and Group High (H) (ESR ≥ 61 mm). Method agreement was assessed by Bland-Altman analysis and Passing-Bablok regression.

**Results:**

Analyser iSED has shown better comparability with Westergren method (bias 0.0 (95%Cl -1.4 to 1.5) range than Roller 20 PN (bias = - 6.4 (95%Cl - 7.1 to -5.7) in the whole measuring. For Roller 20 PN, Passing-Bablok regression has shown constant and proportional difference for Groups L and M, and for iSED only for Group H. Roller 20 PN had lower sensitivity (0.51 (95%Cl: 0.45-0.57) than iSED (0.72 (95%Cl: 0.59-0.80) while they had comparable specificity (> 0.90) and accuracy (≥ 0.80) in comparison with the Westergren method.

**Conclusion:**

Both analysers are not comparable with the Westergren method and should not be used interchangeably.

## Introduction

Erythrocyte sedimentation rate (ESR) is a widely used assay which is still among the top 25 most common laboratory tests (*1*). Because of its simplicity and use for monitoring inflammatory, autoimmune and malignant diseases, it still has clinical use. Many physiological and pathophysiological causes can increase the ESR values which make this test nonspecific, *e.g.*, in pregnancy, for women who have menstruation, at an older age, arthritis and inflammatory bowel disease (*2*). The “gold standard” for the determination of ESR is the Westergren method which is standardized but time-consuming (*3*). Westergren method uses whole blood sample that is diluted with a liquid citrate anticoagulant (4:1) and the value of ESR is determined after one hour in a vertically placed tube. Furthermore, for performing ERS with the Westergren method larger sample volume is needed. In order to shorten the time of the ESR measurement, there are modified Westergren methods that have certain modifications (*e.g.*, shorter turnaround time, use of non-diluted samples) and alternate methods that are based on a different methodology. The survey conducted by International Council for Standardization in Haematology (ICSH) indicate that 2/3 of all laboratories worldwide use modified or alternate ESR test method (*3*). These alternate ESR methods can be based on photometric rheology which measures Rouleaux formation (transmitted or reflexed light intensity depending on the duration of erythrocyte aggregation) or centrifugation (*3*). The obtained results by the alternate method are mathematically transformed into values comparable with the Westergren method (*3*, *4*).

Not only does the alternate methods reduce the turnaround time to 30 minutes or less for measurement of ESR but they also have more advantages, *e.g.* reducing possibility of a human error (the possibility of misreading ESR values or wrong transcript on the laboratory report). Furthermore, they reduce the cost of the blood collection devices and a volume of blood required for analysis, because the ESR analysers use ethylenediaminetetraacetic acid (EDTA) whole blood. The use of EDTA blood also reduces the possibility of errors, due to the dilution of the sample with citrate anticoagulants and decrease the risk of exposure of laboratory technician to possible infectious blood pathogens, during sample handling (*2*). Several automated or semi-automated methods are available for estimating ESR values and consequently, various ESR analysers have been developed (*3*). Over the last 20 years there were numerous verification and comparison studies of several automated analysers with Westergren method. Several studies have already investigated analytical performances of analysers iSED (Alcor Scientific, Smithfield, SAD) and TEST1 (Alifax S.p.A, Polverara, Italy) (*5*-*7*). Analyser TEST1 is widely used for the measurement of ESR, while the newly developed Roller 20PN (Alifax S.p.A, Polverara, Italy) adheres to similar technology but compared to the earlier models has slightly different performance of measuring ESR (*8*). Moreover, because of the altered methodology of automated ESR analysers that are increasingly used in routine practice instead of the Westergren method, the verification of these methodologies is highly recommended by ICSH. Additionally, it is questionable whether the obtained ESR results of these automated ESR analysers are even comparable to the Westergren method. Furthermore, the performance and comparison of two automated ESR analysers, Roller 20 PN and iSED, have not yet been investigated.

The aim of this study was therefore to perform the verification of the precision and accuracy of two automated ESR analysers: Roller 20 PN and iSED and to compare these two automated analysers with Westergren method and with one another.

## Materials and methods

### Subjects

This verification study was done from September to December 2017, in the Department of medical laboratory diagnostics in University Hospital “Sveti Duh”. All patients for whom tests for ESR and complete blood count have been ordered were included in this study and their leftover blood samples were used for analysis. The exclusion criteria were not used because we wanted to include as many participants available to cover a wider range of ESR values. Furthermore, all included patients represent a representative population of our laboratory. For each patient two blood tubes were used: one 1,6 mL tube with 3,8% sodium citrate (Kima, Piove di Sacco, Italy) and other 3 mL tube with K_2_EDTA (Kima, Piove di Sacco, Italy). Samples were analysed within 4 hours from arrival to the laboratory. The study was done with the approval of the hospital Ethics Committee for using leftover blood samples.

### Methods

Both analysers iSED and Roller 20 PN are based on alternate ESR methodology which measures aggregation of red blood cells, and then transforms results to Westergren values. Analyser iSED takes 100 µL of sample directly from EDTA blood tube, after which the aggregation of erythrocytes is measured in microflow cell by optical detector. Analyser Roller 20PN withdraws 175 µL also from EDTA blood sample and the aggregation of erythrocytes is measured in the capillary by photometric method. The maximum number of samples that can be analysed simultaneously are 20 for both analysers.

Performance verification of Roller 20 PN and iSED was based on the Clinical Laboratory Standard Institute (CLSI) H02-A5 and CLSI EP15-A2 documents (*9*, *10*). For determination of precision control samples were used for both automated analysers. For Roller 20 PN, Latex control samples were used (REF SI 305.300-A, Lot N. 1828 C, Alifax S.p.A., Polverara, Italy) in three concentration levels: Level 2 (6-11 mm), Level 3 (15-22 mm) and Level 4 (56-74 mm). For iSED, Seditrol quality control samples were used (REF DSCO6, Lot #27, Alcor Scientific, Smithfield, USA) in two concentration levels: Level 1 (2-16 mm) and Level 2 (38-90 mm). Between-run precision was determined in triplicate for 5 consecutive days. For within-run, control samples and leftover blood samples (6 samples for Roller 20PN and 7 samples for iSED) were analysed 20 times in a row in one day. Between- and within-run precision was expressed as coefficient of variation in percentage (CV%). For Roller 20PN, the obtained CVs% were compared to declared CVs% by manufacturer. For analyser iSED the manufacturer did not declare the criteria for within- and between run CV%. The criteria for within and between-run, for iSED, were calculated from 1 standard deviation (SD) and the mean which were declared by the manufacturer for control samples.

For determination of accuracy and method comparison, each sample was analysed in parallel with Westergren method and on automated analysers Roller 20 PN and iSED. Moreover, EDTA samples were analysed in triplicate on automated analyser. For better comparison of automated analysers with Westergren method we divided data into three groups as it was recommended by ICSH: i) Group L (as low) with value of ESR ≤ 20 mm, ii) Group M (as medium) with value ESR 21-60 mm and iii) Group H (as high) with ESR value ≥ 61 mm. The data was not divided by relevant reference interval (RI) because in our department there are more different RI according to the age and gender. Sensitivity, specificity, and accuracy were also determined for both analysers. For this calculation, values were dichotomized into positive (above the upper value of the Group L) and negative (within the values in Group L), with a Westergren method as a reference.

### Statistical analysis

For statistical analysis MedCalc Statistical Software version 14.8.1 (MedCalc Software bv, Ostend, Belgium) and Microsoft Office Excel 2010 (Microsoft, Washington, USA) were used. Bland Altman analysis and Passing Bablok regression were used for comparison of two analysers with Westergren method (P < 0.05 was considered as a level of significance). Where cusum test showed significant deviation from linearity (P < 0.05), the Passing-Bablok regression was not performed for the data. Sensitivity and specificity were determined by Diagnostic and Agreement Statistics (DAG_Stat) (*11*).

## Results

The obtained CVs% for between- and within-run precision on control samples for both analysers are presented in Table 1. The wider acceptable range defined by the manufacturer for Seditrol quality control sample Level 1 for analyser iSED may be the reason for higher CV%. Moreover, 14 of 15 measurements were within the range of 8-13 mm, and only one measurement was 4 mm which was still within the defined range declared by the manufacturer (2-16 mm) but significantly alters CV%. Within-run precision CVs% obtained from leftover patients’ samples, for Roller 20PN, in higher ESR values (27-90 mm) were lower than 10% (Table 2). On the other hand, within-run precision CV% for iSED determined on 7 different leftover patients’ samples with ESR values 8-90 mm was between 10.3-17.1% (Table 2).

**Table 1:** Within- and between-run precision for ESR automated analysers Roller 20 PN and iSED

**Control sample (declared range by manufacturer in mm)**	**Within-run precision CV%**	**Between-run precision CV%**	**Criteria for within-run precision defined by manufacturer**	**Criteria for between-run precision defined by manufacturer**
**Roller 20 PN**				
**Latex control samples**				
Level 2 (6–11)	8.8	5.2		
Level 3 (15–22)	7.3	3.3	5.7	5.1
Level 4 (56–74)	5.7	2.6		
**iSED**				
**Seditrol quality control samples**				
Level 1 (9 ± 7)	28.7	2.9	38.9	38.9
Level 2 (64 ± 26)	5.3	7.8	20.3	20.3
CV – coefficient of variation. ESR – erythrocyte sedimentation rate.				

**Table 2:** Within-run precision for automated analysers Roller 20 PN and iSED on leftover patients’ blood samples with different values of ESR

**Patients’ samples**	**Mean value (mm)**	**Coefficient of variation CV%**	**Acceptance criteria for within-run precision defined by manufacturer**
**Roller 20 PN**			
Sample A	16	15.7	
Sample B	22	10.2	
Sample C	27	4.1	5.7
Sample D	40	4.0	
Sample E	81	16.1	
Sample F	90	2.2	
**iSED**			
Sample G	8	17.1	
Sample H	14	12.4	38.9
Sample G	17	15.4	
Sample K	24	13.1	
Sample L	30	12.1	
Sample M	72	10.7	20.3
Sample N	90	10.3	
CV – coefficient of variation. ESR – erythrocyte sedimentation rate.			

**Figure 1:**
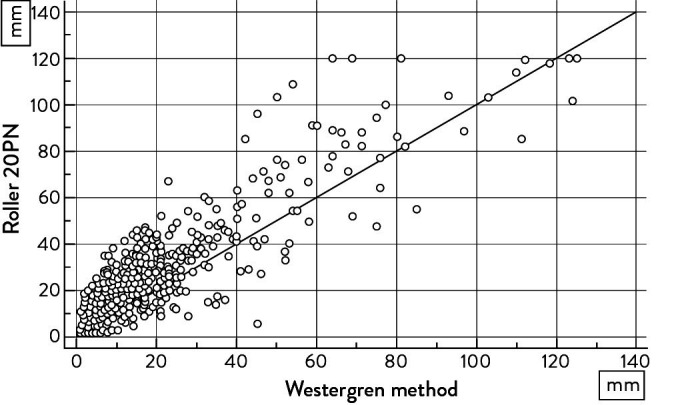
Scatter plot showing relationship between analyser Roller 20PN and Westergren method for estimation ESR. ESR – erythrocyte sedimentation rate.

**Figure 2:**
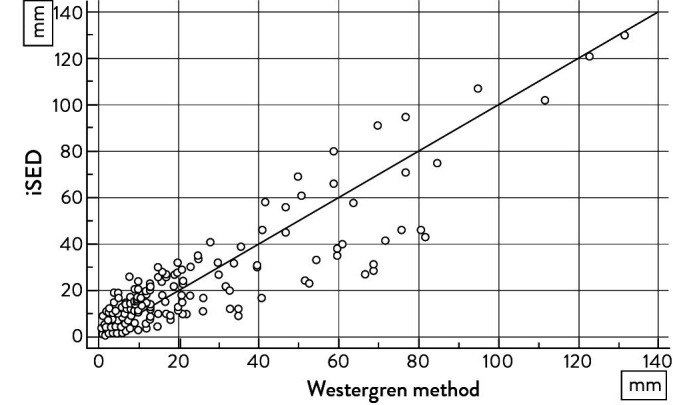
Scatter plot showing relationship between iSED and Westergren method for estimation ESR. ESR – erythrocyte sedimentation rate.

**Figure 3:**
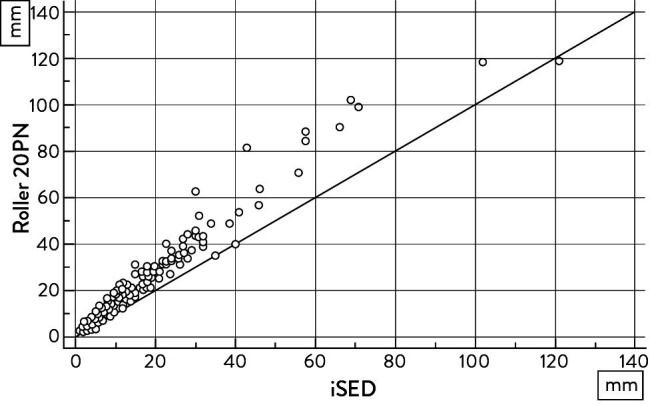
Scatter plot showing relationship between iSED and Roller 20PN for estimation ESR. ESR – erythrocyte sedimentation rate.

For method comparison for Roller 20PN and iSED with Westergren method, a total of 752 and 213 subjects were included. Also, a total of 196 subjects were included for method comparison between analysers Roller 20PN and iSED. The discrepancy in the number of included subjects for method comparison is due to the difference in time period for verification of each analyser. Analyser iSED was verified in a shorter time period than analyser Roller 20PN, and during that short period compared with Roller 20PN and Westergren method. Bland-Altman analysis showed a negative mean bias for comparison Roller 20PN with Westergren, while for comparison iSED with Westergren method the mean bias was not statistically significant in the whole measuring range (Table 3). Moreover, Passing-Bablok regression for Roller 20PN showed a constant and proportional difference from the Westergren method for groups L and M, but for analyser iSED there was a constant and proportional difference only for group H (Table 3). Scatter graphs provide insight into the relationship of data measured with different methods (Figure 1-3).

**Table 3:** Method comparison between Roller 20PN or iSED with Westergren method, divided into three groups, according to the ESR values

**Patient group**	**N**	**Mean bias (95%CI)**	**Cusum test**	**Intercept (95%CI)**	**Slope (95%CI)**
**Roller 20 PN**					
The whole range of ESR values	752	- 6.4 (- 7.1 to - 5.7)	P < 0.010	/	/
Group L	597	- 6.3 (- 6.8 to - 5.7)	P = 0.110	- 1.4 (- 2.3 to - 1.0)	2.1 (2.0 to 2.3)
Group M	125	- 7.2 (- 9.9 to - 4.5)	P = 0.080	- 23.0 (- 36.0 to - 15.6)	2.0 (1.7 to 2.4)
Group H	30	- 6.2 (- 14.0 to 1.6)	P = 0.130	- 3.2 (- 72.5 to 31.9)	1.1 (0.7 to 1.9)
**iSED**					
The whole range of ESR values	213	0.0 (- 1.4 to 1.5)	P = 0.020	/	/
Group L	157	- 2.8 (- 3.6 to - 2.0)	P = 0.220	- 1.0 (- 2.4 to 0.4)	1.6 (1.4 to 1.8)
Group M	39	4.8 (0.2 to 9.4)	P = 0.770	- 18.1 (- 37.4 to - 4.2)	1.5 (1.0 to 2.0)
Group H	17	15.2 (4.5 to 25.9)	P = 0.580	- 89.7 (- 208.2 to - 42.5)	1.8 (1.3 to 3.4)
Group L with ESR values ≤ 20 mm; Group M with ESR values 21-60 mm and Group H with ESR values > 61 mm. P < 0.05 was significant deviation from linearity for cusum test. ESR – erythrocyte sedimentation rate. 95%Cl - 95% confidence interval.					

**Figure 4:**
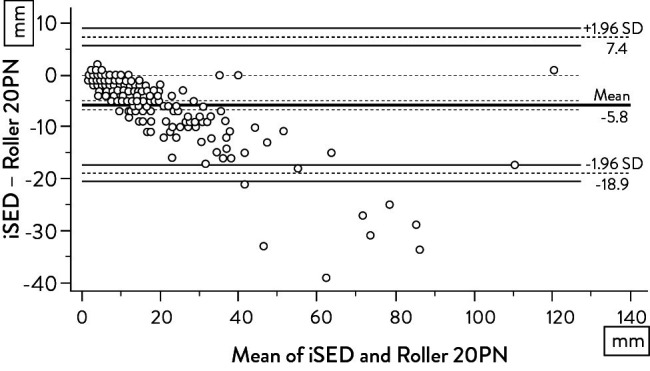
Bland Altman plots showing the difference between iSED and Roller 20PN for estimation ESR. Solid line (Mean) represents mean difference and dotted lines around Mean represents 95% confidence interval. Dashed lines represent ± 1.96SD with associated 95% confidence interval (dashed lines). ESR – erythrocyte sedimentation rate. SD - standard deviation.

**Figure 5:**
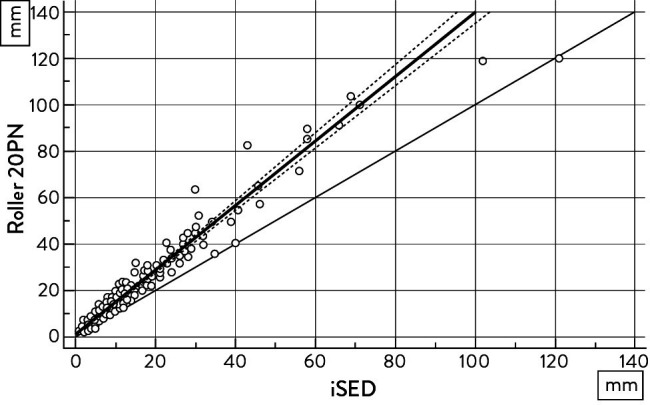
Passing Bablok scatter diagram for comparison of iSED and Roller 20PN analysers for estimation ESR. Solid line represents regression line and dotted lines represent 95% confidence interval. ESR – erythrocyte sedimentation rate.

For comparison of two analysers, Bland-Altman analysis determined statistically significant negative mean bias between Roller 20PN and iSED (Figure 4). Constant and proportional difference was evident in the comparison of Roller 20 PN and iSED. Equation of Passing Bablok regression is: y = 0.4 (95%CI = 0.9 to 0.1) + 1.4 (95%CI = 1.4 to 1.5) x (Figure 5).

Roller 20 PN had lower sensitivity compared to iSED, while they had comparable specificity and accuracy (Table 4).

**Table 4:** Calculated sensitivity and specificity for two ESR analysers, Roller 20 PN and iSED

	**Roller 20PN**	**iSED**
**Sensitivity**	0.51 (95%CI = 0.45 to 0.57)	0.72 (95%CI = 0.59 to 0.83)
**Specificity**	0.96 (95%Cl = 0.94 to 0.98)	0.92 (95%CI = 0.87 to 0.96)
**Accuracy**	0.80 (95%CI = 0.77 to 0.83)	0.86 (95%CI = 0.81 to 0.91)

## Discussion

Our study demonstrates that the overall precision on control and patients samples was better in the higher range of ESR values. Because of the different methodology of measurement for ESR, the comparability of Westergren method and both automated analysers indicate significant discrepancy, but both analysers demonstrated great specificity (> 90%). Interestingly, comparison between Roller 20PN and iSED showed significant bias despite the similar methodology.

Verification of automated analyser iSED was performed by several studies (*6*, *7*, *12*). Schapkaitz *et al.* and Lapić *et al.* results showed better precision for control samples but similar CVs% were estimated with patient samples. Plebani *et al.* indicated different kinetic properties of control samples and fresh blood samples, which might be the cause of the diversity of CV% precision (*13*).

All three studies showed similar results for comparison of iSED with Westergren method, with the exception of the negative bias for lower ESR values, which was observed in our study and not in others (*6*, *7*, *12*). This disagreement may be due to some patient characteristics and sample diversity used for comparison.

By comparing analyser TEST1 with Westergren method, Lapić *et al.* showed presence of negative bias, constant and proportional difference for whole measuring range, which agrees with our results. The authors also divide results in three groups by ESR values determined by Westergren method, but in neither of this groups the constant or proportional difference were found for comparison TEST1 with Westergren method (*6*). The discrepancy of those results with ours for method comparison may be caused by comparing similar but not the same analysers (TEST1 and Roller 20 PN) with Westergren method and dividing groups by different cut-off values. Our study showed that Roller 20 PN overestimates ESR values throughout whole measuring range values and iSED overestimates ESR values only in patients with ESR ≤ 20 mm. Overestimation of ESR values in patients with already higher ESR values is not clinically significant, but because of the lack of agreement between those two ESR automated analysers and Westergren method, the estimation of new reference interval would be recommended. Furthermore, by obtaining the new reference interval (whose value would be used as cut-off), the sensitivity and specificity would be different.

Moreover, similar to our findings, Lapić *et al.* have implied existence of difference in ESR values which were obtained by comparison of two analysers (iSED and TEST1). The difference between analysers that are based on a similar methodology may be because of different time frame of pre-analysis sample mixing. Despite the similarity of methods, the whole analysis process is not yet harmonized completely (*7*).

The limitation of this study is that it included only a minor proportion of the samples with ESR values higher than 60 mm. Further studies are needed to confirm our findings in the range of elevated ESR values. Moreover, we are aware of potential interference of low haematocrit and concentrations of positive acute phase proteins (fibrinogen) and potential physiological and pathophysiological conditions of patients that may affect analysis of ESR values. The more extensive verification which would include the investigation of potential interferences in order to upgrade the quality measurement of ESR is highly warranted. Another limitation of this study was that the number of included samples for verification of Roller 20PN and iSED was disproportional.

Implementation of automated measurement of ESR have many advantages, such as the use of the control samples for better monitoring of measurement quality, reduction of preanalytical and analytical errors of manual measurement and many more which were mentioned previously in this study. Before replacing a test used in the routine with a test based on different methodology, the clinicians need to be informed about the potential effect on the test results and about interpretation on which future clinical decisions are going to be based.

## Conclusion

Both analysers, Roller 20PN and iSED, are not comparable with the Westergren method and should not be used interchangeably. Overall, the disagreement with Westergren method is less pronounced for iSED analyser.
